# Nitroglycerin for Management of Retained Placenta: A Multicenter Study

**DOI:** 10.1155/2012/321207

**Published:** 2012-05-22

**Authors:** Maria Bullarbo, Hans Bokström, Håkan Lilja, Elisabeth Almström, Nina Lassenius, Agneta Hansson, Erling Ekerhovd

**Affiliations:** ^1^Department of Obstetrics and Gynecology, Södra Älvsborg Hospital, 501 82 Borås, Sweden; ^2^Department of Obstetrics and Gynecology, Sahlgrenska University Hospital, 413 45 Gothenburg, Sweden; ^3^Department of Obstetrics and Gynecology, NÄL Hospital, 461 85 Trollhättan, Sweden; ^4^Department of Obstetrics and Gynecology, Vrinnevi Hospital, 603 79 Norrköping, Sweden; ^5^Department of Obstetrics and Gynecology, Central Hospital of Karlstad, 651 85 Karlstad, Sweden; ^6^Fertility Clinic Sør, Telemark Hospital, 3901 Porsgrunn, Norway

## Abstract

The primary aim was to determine if sequential administration of oxytocin and nitroglycerin is effective for management of retained placenta when performed by obstetricians with no experience of the method. Secondary aims were to examine possible adverse effects of nitroglycerin. One hundred and five women with retained placenta were randomly selected to receive either 1 mg nitroglycerin or placebo tablets sublingually if intravenous oxytocin had failed to expel the placenta. At two of the hospitals some of the midwives were familiar with the use of nitroglycerin. The other midwives and all the participating obstetricians had no clinical experience of the method. In the treatment group, detachment of placenta following nitroglycerin occurred in 37.3% of the women compared to 20.4% in the placebo group (*P* = 0.056). In the two hospitals with some experience of the method, placenta was removed in 9 of 19 (47.4%) women in the nitroglycerin group compared to 3 of 17 (15.0%) women in the placebo group. No adverse effects of clinical importance were registered. Although the difference between the two groups did not reach statistical significance, the higher success rate in the two hospitals with some experience could indicate that clinical experience is of importance in order to achieve placental detachment.

## 1. Introduction

Retained placenta complicates up to 2.0–3.3% of all vaginal deliveries [1, 2]. The most common cause of retained placenta has been shown to be a failure of retroplacental myometrial contractions [3]. Without immediate treatment, women are at high risk of hemorrhage. The delivery of oxytocin to the retroplacental myometrium via the umbilical vein or intravenously is often applied to induce myometrial contractions and placental detachment. The WHO guidelines for management of postpartum hemorrhage and retained placenta recommend injection of saline-diluted oxytocin into the umbilical vein [4]. However, a recently published large randomized study concluded that this method has no clinically significant effect [5]. Thus, manual removal of the placenta under general or regional anesthesia is usually needed.

 Alternatively, when oxytocin fails, it has been shown that the sequential administration of oxytocin and sublingual nitroglycerin is efficient for the management of retained placenta [6, 7]. Nitroglycerin relaxes smooth muscle cells by releasing nitric oxide and is regularly used to induce prompt uterine relaxation in cases of obstetric emergency [8].

 A recent Cochrane review concluded that sublingual nitroglycerin seems to both reduce the need for manual removal of placenta and blood loss during the third stage of labour when compared to placebo [9]. However, it was also emphasized that further trials are needed to confirm its clinical role and safety.

 A very high success rate for management of retained placenta was reported following sequential administration of oxytocin and nitroglycerin when performed by obstetricians who were experienced in this method [6, 7]. However, in an everyday clinical setting we noticed that many obstetricians with little or no experience in this method often failed in their attempts to remove the placenta. The primary aim of the present study was therefore to determine if oxytocin in combination with nitroglycerin is effective in the management of retained placenta when performed by inexperienced obstetricians. Secondary aims were to examine hemodynamic effects, blood loss, and side effects following sublingual administration of nitroglycerin and to identify factors that could have a negative effect on placental detachment.

## 2. Materials and Methods

This was a prospective double-blind randomized controlled multicenter study that was carried out at five Swedish hospitals between October 2008 and July 2010. Two of the hospitals (hospitals A and B) were university hospitals with approximately 4100 and 6400 deliveries per year, respectively, while the three other hospitals were referral hospitals (hospitals C, D, and E) with 2100 to 3200 deliveries per year. At the two university hospitals some of the midwives were familiar with the use of nitroglycerin for management of retained placenta. None of the midwives at the three other hospitals or any of the obstetricians involved in the study had any clinical experience of the method. All participating obstetricians and midwives received written information about the study. Inclusion criteria were uncomplicated singleton pregnancy with spontaneous vertex delivery of a healthy child at term. Exclusion criteria were serious maternal disease, maternal age less than 18 years, blood loss more than 600 mL, uterine malformation, or suspected placental accreta. Thus, women with previous Cesarean section were not excluded from participating in the study. The study was approved by the medical ethics committees of the participating hospitals.

 All pregnant women who planned to give birth at any of the five hospitals received written as well as oral information about the study several weeks before expected delivery. According to the study protocol, if the placenta remained undelivered 30 minutes after child birth, a slow push of 10 IU (16.6 *μ*g) oxytocin (Syntocinon, Novartis, Täby, Sweden) was administered intravenously to promote uterine contractions and placental detachment. Five minutes after injection of oxytocin, controlled cord traction was performed once more to facilitate placental delivery. If the placenta remained undelivered 40 minutes after childbirth the women were asked to participate in the study. They were also informed about possible side effects of nitroglycerin. In addition, intravenous administration of Ringer-acetate was instituted routinely as a prophylactic measure in case of postpartum hemorrhage and all the women were hemodynamically monitored.

 Randomization was carried out at a ratio of 1 : 1 by blocks of 4 (http://www.randomization.com/), and the allocation was performed by means of numbered opaque, sealed envelopes. A nurse who did not participate in the remainder of the study handed out the study medication as needed.

 Following informed consent, the women were allocated to either of the two groups: (1) 1 mg of nitroglycerin tablets (two 0.5 mg tablets) Nitromex, Alpharma AB, Stockholm, Sweden) or (2) two placebo tablets. The tablets were administered sublingually approximately 50 minutes after childbirth. Neither the obstetrician nor the participating women were aware of the agent administered. Five minutes after administration of nitroglycerin or placebo tablets, gentle persistent cord traction was performed once more for a maximum duration of five minutes. The procedure was regarded as successful if the placenta was delivered in the delivery room without the need of operative manual removal under either regional (epidural or spinal) or general anesthesia.

 Maternal blood pressure and pulse rate were measured prior to administration of nitroglycerin or placebo tablets. These measurements were repeated five minutes as well as 15 minutes after administration of the tablets for assessment of possible hemodynamic effects caused by nitroglycerin. In addition, total blood loss during the third stage of delivery was registered. Blood loss was assessed by weight and visual estimation, and in some cases also combined with measurement of collected blood poured into a measuring jar. The women also completed a questionnaire regarding possible side effects of nitroglycerin. In addition, medical records were routinely checked in order to identify factors that could adversely affect the outcome of the procedure.

### 2.1. Statistics

A sample size of 114 women (57 in each group) was calculated to yield a power of 90% at the 5% significance level when assuming a success rate of 50% in the treatment group compared to 20% in the placebo group. Some withdrawals from the study were expected and therefore a sample size of 120 women was decided. Continuous variables between the treatment group and the placebo group were analyzed by using the Mann-Whitney *U* test, while the Chi-square test was used for analysis of dichotomous variables. For comparison between groups the Mantel-Haenszel Chi-square test was used for ordered categorical variables and the Chi-square test was used for non-ordered categorical variables. The 95% confidence interval was used for assessment of relative risk for dichotomous variables. Change from baseline variables within treatment groups was analyzed by using the Wilcoxon signed rank test. For categorical variables *n* (%) is presented and for continuous variables Mean, SD, median, min, and max are presented. Significance tests were two-sided and conducted at 5% significance level.

## 3. Results

One hundred twenty women agreed to participate in the study. However, two women were excluded since only one tablet (0.5 mg) of nitroglycerin by mistake was administered. In addition, spontaneous placental detachment occurred in three women immediately before administration of tablets, while in one case umbilical cord disruption occurred before administration of tablets. Furthermore, in nine women surgical removal of placenta was performed initially due to the preference of the obstetrician on duty despite the fact that the women had given their informed consent to participate. Thus, the total number of patients in the intention to treat group (ITT) was 111 (all randomized patients, including 4 patients who did not receive study medication and 2 patients who only received 0.5 mg nitroglycerin), and the number of patients in the per protocol group (PP) was 105 (randomized patients who all received study medication, i.e., 1 mg nitroglycerin or placebo tablets).

 There were no differences between the two groups regarding maternal age, parity, or site of study (Table 1). For the intention to treat group (ITT) detachment of placenta following sublingual administration of nitroglycerin occurred in 36.4% of the women compared to 23.2% in the placebo group (*P* = 0.13) (Table 2). The corresponding results for patients per protocol group (PP) were 37.3% and 20.4% (*P* = 0.056), respectively (Table 3). In the two hospitals with some experience of the method placenta was successfully removed in 9 of 19 (47.4%) women following treatment with nitroglycerin compared to 3 of 17 (15.0%) women in the placebo group. The difference between the treatment groups was 32.4% (95% CI −0.5; 58.8).

**Figure 1:**
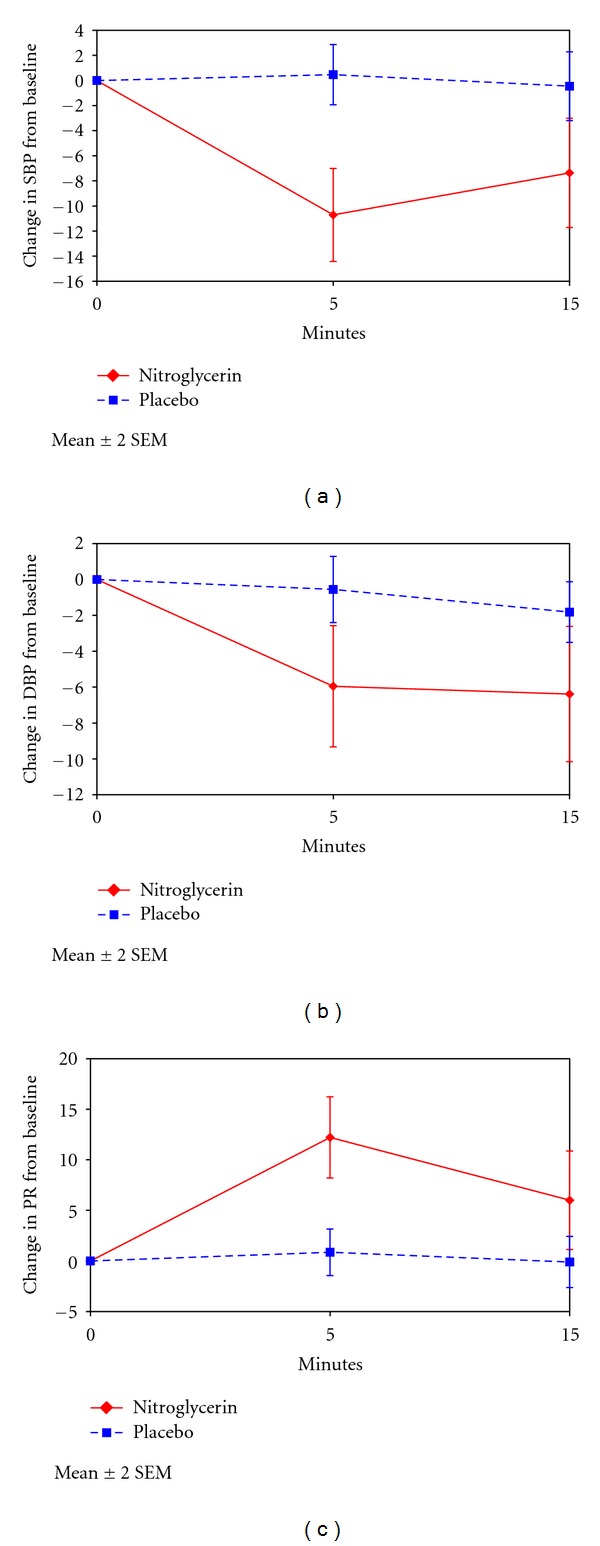
Systolic blood pressure (SBP), diastolic blood pressure (DBP), and pulse rate (PR) at baseline, 5 and 15 minutes after administration of either nitroglycerin or placebo tablets. All parameters showed differences of statistical significance between the groups (*P* < 0.05) following administration of the tablets.

 A significant difference in blood pressure as well as pulse rate between the nitroglycerin group and the placebo was registered at 5 and 15 minutes after administration of tablets (Figure 1). Maximum drop in blood pressure was measured 5 minutes after nitroglycerin had been given. However, in all women who received nitroglycerin hemodynamic changes were moderate and considered not to be of clinical importance. There was no difference between the groups regarding total blood loss or blood loss more than 1000 mL. No complications of clinical importance due to nitroglycerin were registered.

 Headache and palpitations were relatively common in both groups (Table 4). However, reported maternal side effects were only of minimal or moderate intensity with a maximum of 6 on a VAS scale from 1 to 10. No statistical difference was registered between the two groups regarding frequency of side effects or intensity of side effects.

## 4. Discussion

In the present study successful release of placenta occurred in 37.3% of the women with retained placenta following sequential administration of oxytocin and nitroglycerin. The corresponding figure in the two hospitals with some experience of the method was 47.4%. Although none of these figures reached statistical significance compared to placebo, the higher success rate in the two hospitals with some experience could indicate that clinical experience is of importance in order to achieve placental detachment.

 Several descriptive and retrospective studies have examined the use of nitroglycerin for the release of retained placenta [7, 10–14]. However, only one randomized study has previously evaluated the efficiency of sequential administration of oxytocin intravenously and nitroglycerin tablets [6]. In that study, retained placenta was successfully released in all 12 women following administration of nitroglycerin compared to only 1 of 12 women in the placebo group [6]. In addition, in a chart review of 24 women with retained placenta, successful delivery of the placenta occurred in 21 of the women following sequential treatment with intravenously administered oxytocin and 1 mg of nitroglycerin tablets administered sublingually [7]. However, in both studies only obstetricians who were experienced in the method were involved in the attempts to remove the placenta. Thus, it seems likely that clinical experience is essential in order to accomplish a high success rate.

 Three main causes of retained placenta have been identified: (1) placenta adherens (81%); (2) trapped placenta (13%); partial placenta accreta (6%) [15]. Placenta adherens is due to inadequate myometrial contractions to separate the placenta [3]. Trapped placenta signifies a detached placenta that is trapped behind a closed uterine cervix, while placenta accreta indicates that an area of the placenta is firmly attached to the myometrium [15]. In the present study ultrasonography was not performed routinely to distinguish between the different types of retained placenta. However, none of the women was diagnosed with placenta accreta.

 A significant drop in both systolic and diastolic blood pressure as well as a significant increase in pulse rate was registered following administration of nitroglycerin (Figure 1). However, in all women the hemodynamic effects were transient and nitroglycerin-induced uterine relaxation did not aggravate vaginal hemorrhage. These findings confirm previous studies which have shown that sublingual administration of 1 mg nitroglycerin in women with retained placenta and normal blood loss is a safe procedure [6, 7].

 Many of the women reported side effects, especially headache and palpitations of mild or moderate intensity (Table 4). However, no statistical difference was registered regarding frequency and intensity of side effects between the nitroglycerin and the placebo group. The high number of reported side effects may be due to the fact that the women were informed about possible adverse effects of nitroglycerin before giving informed consent.

 Sequential treatment with oxytocin and nitroglycerin is supposed to initiate uterine contractions followed by transient uterine relaxation, thereby promoting placental detachment. In the present study three factors that could possibly affect the outcome of the method negatively were identified following discussions with midwives and cross-checking of the medical records: (1) oxytocin infusion in the third stage was continued after administration of nitroglycerin; (2) breastfeeding of the newborn child/nipple stimulation was continued after administration of nitroglycerin; (3) abdominal uterine massage to promote contractions was performed after administration of nitroglycerin. Clearly, all these factors could possibly counteract the relaxing effect of nitroglycerin.

 One weakness of the present study is that only obstetricians with no experience of the method were involved in the study. On the other hand, the study describes a real-life clinical scenario when a new method is being introduced. It was simply not possible to carry out a multicenter study involving only obstetricians with great experience of the method. The fact that placenta was removed in almost half of the women following administration of nitroglycerin in the two hospitals with some experience of the method is encouraging. Clearly, a large prospective randomized placebo-controlled study performed by obstetricians who have experience of the method would be of interest.

## 5. Conclusion

This study showed an increased release of retained placenta following sequential administration of oxytocin and nitroglycerin compared to oxytocin and placebo. However, the difference in success rate did not reach statistical significance. A tendency that clinical experience of the method could be of importance for removal of placenta was registered. Nitroglycerin did not cause any adverse effects of clinical importance.

## Figures and Tables

**Table 1:** Demographic and baseline characteristics (intention to treat group, ITT).

Variable	Nitroglycerin(*n* = 55)	Placebo (*n* = 56)	*P* value
Age (years)			0.98
Mean (SD)	31.7 (5.0)	31.7 (5.6)	
Median (range)	32 (19; 42)	32 (20; 44)	

Parity n (%)			0.77
1	23 (41.8)	24 (42.9)	
2	20 (36.4)	22 (39.3)	
3	10 (18.2)	7 (12.5)	
4	0 (0.0)	2 (3.6)	
5	2 (3.6)	1 (1.8)	

Site *n* (%)			1.00
A	11 (20.0)	11 (19.6)	
B	13 (23.6)	14 (25.0)	
C	7 (12.7)	8 (14.3)	
D	17 (30.9)	17 (30.4)	
E	7 (12.7)	6 (10.7)	

Blood loss before treatment (mL)*	*n* = 53	*n* = 54	0.94
Mean (SD)	554 (464)	548 (457)	
Median (range)	450 (50; 2400)	450 (0; 2000)	

Time from delivery to administration of nitroglycerin or placebo tablets (minutes)*			0.06
	
Mean (SD)	49.5 (8.9)	46.3 (7.5)	
Median (range)	50 (27; 70)	46 (31; 62)	

Intention to treat group, ITT: all randomized patients, including 4 patients who did not receive study medication and 2 patients who only received 0.5 mg nitroglycerin. *Four patients who did not receive study medication are not included.

**Table 2:** Clinical data for management of retained placenta by treatment group (ITT).

Variable	Nitroglycerin (*n* = 55)	Placebo (*n* = 56)	*P* value	Relative risk (RR) 95% CI
Placental detachment				
*n* (%)	20 (36.4)	13 (23.2)	0.13	1.57 (0.87; 2.83)

Blood loss after treatment (mL)	*n* = 51	*n* = 54		
Mean (SD)	465 (463)	560 (468)	0.30	
Median (range)	250 (50; 2200)	400 (0; 1800)		

Total blood loss (mL)	*n* = 51	*n* = 54		
Mean (SD)	1018 (612)	1098 (658)	0.52	
Median (range)	900 (200; 2500)	950 (150; 3000)		

Total blood loss ≥1000 mL				
*n* (%)	24 (43.6)	26 (46.4)	0.77	0.94 (0.62; 1.42)

Intention to treat group, ITT: all randomized patients, including 4 patients who did not receive study medication and 2 patients who only received 0.5 mg nitroglycerin.

**Table 3:** Clinical data for management of retained placenta by treatment group (per protocol population, PP).

Variable	Nitroglycerin (*n* = 51)	Placebo (*n* = 54)	*P*-value	Relative Risk (RR) 95% CI
Placental detachment				
*n* (%)	19 (37.3)	11 (20.4)	0.056	1.83 (0.97; 3.46)

Blood loss after treatment (mL)	*n* = 48	*n* = 53		
Mean (SD)	474 (470)	569 (468)	0.31	
Median (range)	250.0 (50; 2200)	400 (0; 1800)		

Total blood loss (mL)	*n* = 48	*n* = 53		
Mean (SD)	1020 (604)	1091 (662)	0.58	
Median (range)	925 (200; 2500)	950 (150; 3000)		

Total blood loss ≥1000 mL				
*n* (%)	23 (45.1)	25 (46.3)	0.90	0.97 (0.64; 1.48)

Per protocol population (PP): randomized patients who all received 1 mg nitroglycerin or placebo tablets.

**Table 4:** Maternal side effects following medical treatment (ITT group).

Side effect	Nitroglycerin(*n* = 55)	Placebo (*n* = 56)	*P* value
Headache (VAS scale 1–10)	*n* = 34	*n* = 33	
Mean (SD)	0.9 (1.6)	0.5 (1.3)	0.08
Median (range)	0.0 (0; 6)	0.0 (0; 6)	

Palpitations (VAS scale 1–10)	*n* = 29	*n* = 32	
Mean (SD)	0.4 (1.1)	0.2 (0.5)	0.36
Median (range)	0.0 (0; 5)	0.0 (0; 2)	

Intention to treat group, ITT: all randomized patients, including 4 patients who did not receive study medication and 2 patients who only received 0.5 mg nitroglycerin.
